# A chaotic digital signature algorithm based on a dynamic substitution box

**DOI:** 10.1038/s41598-024-83943-x

**Published:** 2025-01-19

**Authors:** Rolando Flores-Carapia, Víctor Manuel Silva-García, Manuel Alejandro Cardona-López, Miguel Gabriel Villarreal-Cervantes

**Affiliations:** 1 Centro de Innovación y Desarrollo Tecnológico en Cómputo, Instituto Politécnico Nacional, 07738 CDMX, México; 2 Centro de Investigación en Computación, Instituto Politécnico Nacional, 07738 CDMX, México; 3 Escuela Superior de Ingeniería Mecánica y Eléctrica, Instituto Politécnico Nacional, 07738 CDMX, México

**Keywords:** Chaos, Diffie-Hellman Protocol, Digital Signature, Number Pi, Substitution Box, Applied mathematics, Computational science, Computer science, Information technology

## Abstract

Given the large volumes of sensitive information transmitted over the Internet, digital signatures are essential for verifying message authenticity and integrity. A key challenge is minimizing computationally intensive operations, such as modular inverses, without compromising security. In this research, we propose the DSADH$\pi$ algorithm, which introduces a confusion step directly into the signature itself, rather than only applying it to the message, using a dynamic substitution box. It is generated with the number pi and changes with each signing. In addition, to enhance security, this work uses a 2048-bit prime, double the length frequently used. This proposal induces chaotic behavior in the signature, making it highly sensitive to any changes in the signer’s private key or message content, thereby enhancing authentication and integrity verification. Moreover, the proposed algorithm computes a single multiplicative modular inverse during verification and none during signing, unlike other approaches that require inverse computation in both stages. Since the required inverse is for the Diffie-Hellman session key, it always exists and can be precomputed per communication rather than per message. Consequently, DSADH$\pi$ is on average 45 times faster than DSA. Additionally, we introduce a method to assess signature security by constructing images from signature bytes generated by slight changes to the signer’s private key and message. Then, their chaotic behavior is evaluated with cryptographic metrics.

## Introduction

The digital signature^1^is a crucial tool for companies, institutions, governments, and individuals to authenticate documents transmitted over networks^2^. Two well-known examples are the Digital Signature Algorithm (DSA)^3^and the Elliptic Curve Digital Signature Algorithm (ECDSA) that is a variant of the DSA with Elliptic Curve Cryptography^4^. However, these algorithms require the use of a multiplicative modular inverse, which is one of the most computationally expensive operations^5,6^. The time required for the signature and verification processes limits the application range^7^, as it involves computing one inverse for signing and another for verification. Furthermore, since the Euclidean algorithm (one of the methods used to compute inverses) is sequential, it cannot be parallelized, making the double inverse-computation a primary constraint. In terms of security, the generated byte string composing the digital signature relies heavily on modular operations, which alone do not provide sufficient randomness properties^8^, such as a uniform distribution, leading to a lack of these desired characteristics.

In contrast, our proposed method requires only a single inverse computation (that even can be precomputed) and incorporates a substitution box in place of relying solely on modular operations, thereby achieving improved randomness properties in the generated strings of the signature. In this way, Yang et al. improved performance by reducing time consumption, by means of replacing the computationally intensive operations, such as the inverse, with more basic operations to achieve better time efficiency^9^. Similarly, Liu et al. eliminated the costly modular inversion operation from their proposal for both the generation and verification phases^10^. Puthiyidam et al. also employed this approach in the two phases to enhance efficiency in applications such as the Internet of Things^11^. Additionally, some post-quantum algorithms were developed considering the computational demands of modular inverse calculations in low-resource, constrained environments^12^.

While optimizing computational time is important in digital signatures, maintaining security remains a priority. To enhance signature security, Hematpour et al. introduced a confusion step using a chaotic map to generate a substitution box, which was then employed to replace the bytes of the original message, increasing the algorithm’s robustness^13^. Similarly, Alam et al. incorporated a digital signature with RSA encryption to the output of the SHA function^14^, although RSA is known for its high computational cost^15^. On the other way, there is a need for digital signature schemes that integrate chaotic properties^16^while maintaining low execution times, ensuring both efficiency and security^17^.

All the previously mentioned works, including our proposal, are algorithms specifically designed for digital signatures, where the digital signature remains separate from the encrypted message. However, while the primary functions of digital signatures are authentication, integrity, and non-repudiation, they have also been integrated into the encryption process^18–20^. Therefore, it would be beneficial for the digital signature itself to exhibit chaotic properties, enhancing its security and making it more resilient to cryptographic attacks such as the statistical^21^.

To address this issue, in this research, we introduce a novel algorithm, Digital Signature Algorithm with Diffie-Hellman protocol and $\pi$ (DSADH$\pi$), designed for signing messages without the need for inverse calculations during the signing process. The algorithm utilizes the session key from the Diffie-Hellman protocol^22^ and the SHA-512 function. It generates dynamic 8$\times$8 substitution boxes using the digits of the number $\pi$ to introduce an additional layer of confusion to the final signature, extending beyond just the message itself. This approach allows for creating a distinctive S-Box each time a signature is generated, rather than relying on a static one. Moreover, only a single inverse calculation is required, which is guaranteed to exist and is performed solely during the verification process. This feature sets our algorithm apart from others, where the existence of an inverse is not assured, and multiple inverse calculations are often necessary.

Furthermore, we propose a novel method to numerically evaluate the quality of a digital signature by constructing two color images using the resulting bytes of the signature. The first image results from variations in the signer’s private key, highlighting the importance of authentication. The second image is generated by altering the content of the plaintext to sign, emphasizing the importance of integrity. The pixels of these images are then evaluated using parameters such as entropy, correlation, discrete Fourier transform, and the goodness-of-fit test with the $\chi ^2$distribution. However, these measures have not been applied exclusively to digital signatures, as they have been evaluated in conjunction with information from encrypted images^23^.

This paper is structured as follows: Supporting elements section presents the necessary background information, including an explanation of the Digital Signature Algorithm (DSA) in a manner analogous to the proposed method, the cryptographic metric for assessing the chaotic degree of information, and relevant properties of the number pi. Proposed digital signature section details the generation of substitution boxes from a permutation and introduces the proposed digital signature algorithm. In Experimentation and results section, we describe the experiments conducted, including the generation of images from different signatures and their evaluation using cryptographic metrics. Results analysis and discussion section provides an analysis of the results, while Conclusion section concludes the paper.

## Supporting elements

### Digital signature algorithm (DSA)

This section provides a comprehensive overview of the Digital Signature Algorithm (DSA). To begin, we will elucidate the constituent elements integral to this process, where the signer and verifier are denoted as *A* and *B*.

#### Parameters and keys generation

1. Primes: Two primes, denoted as *p* and *q*, are chosen in such a way that $p-1$ is divisible by *q*.
2. Generator: A number $\alpha$ is designated as the generator if $\alpha ^{(p-1)/q} \pmod {p} \not \equiv 1$ for all the prime factors *q* of $p-1$.
3. Hash function: A SHA function *H*, such as SHA3-512, is selected.
4. Private keys: Two numbers, denoted as $a_A$ and $a_B$, are selected as the private keys for users *A* and *B* respectively.
5. Public keys: The users possess public keys, denoted as $\beta _A$ and $\beta _B$, calculated as follows: $\beta _A \equiv \alpha ^{a_A} \pmod {p}$ and $\beta _B \equiv \alpha ^{a_B} \pmod {p}$.

#### Procedure for signing a message

1. Randomly select an integer *k*, such that $1< k < q-1$.
2. Compute $\gamma = [\alpha ^k \pmod {p}] \pmod {q}$. If $\gamma =0$, then another *k* is selected to repeat the computation.
3. Compute $\delta = (H(X) + a_A \times \gamma ) \times k^{-1} \pmod {q}$. If $\delta =0$, select another *k* and repeat the computation of the previous steps. Here, *H* represents the chosen SHA function, and *X* is the message to be signed.
4. From this point, A’s signature would be $Sig( X, k) = (\gamma , \delta )$.

#### Procedure for verifying a sign

1. Once *B* has received the message *X*,$\gamma$, and $\delta$, the variable $e_1$ is computed as follows: $e_1 = H(X)\times \delta ^{-1} \pmod {q}$.
2. Additionally, a second variable, $e_2= \gamma \times \delta ^{-1}\pmod {q}$, is computed.
3. With the previous computation of $e_1$ and $e_2$, the verifier B can verify that A signed the message if the following Equation holds true: $[\alpha ^{e_1} \beta _A ^{e_2} \pmod {p}] \pmod {q}= \gamma$.

### Encryption metrics

Below are the instruments commonly employed to quantify the level of disorder within image data. The images in question were constructed using blocks of signatures generated through the proposed algorithm in this study.

#### Correlation coefficient

The correlation coefficient *r*, quantifies the strength of the lineal relationship between two variables^24^. In this work, we examine the interrelationships among pixel values within an image. Initially, a random sample of size *n*is selected from the image under consideration. Subsequently, the correlation between the first variable representing the randomly chosen pixel values and the second variable with their corresponding adjacent pixels is assessed in three directions: horizontal, vertical, and diagonal^25^.

Let $0 \le x_{i,c} \le 255$ denote the intensity of the $i-$th randomly chosen pixel, where $1 \le i \le n$ and *c* denotes the color channel (red, green, or blue). The adjacent pixels values are referred to as $h_{i,c}$, $v_{i,c}$, $d_{i,c}$ for horizontal, vertical, and diagonal direction, respectively, with the same intensity range $0 \le h_{i,c}, v_{i,c}, d_{i,c} \le 255$.

For instance, the correlation coefficient for the horizontal direction and the red color channel is expressed using Equation (1).

1 $$\begin{aligned} r_{x_rh_r}=\frac{1/n\sum _{i=1}^n(x_{i,r}-\bar{x}_r)(h_{i,r}-\bar{h}_r)}{\sqrt{1/n\sum _{i=1}^n(x_{i,r}-\bar{x}_r)^2 \times 1/n\sum _{i=1}^n(h_{i,r}-\bar{h}_r)^2}} \end{aligned}$$

where the arithmetic mean of $\bar{x}_r$ is defined in Equation (2)

2 $$\begin{aligned} \bar{x}_r=\frac{1}{n}\sum _{i=1}^nx_{i,r} \end{aligned}$$

#### Entropy

Entropy *H*quantifies the level of information disorder within encrypted messages, in our context to color images^26,27^. Since images are composed of pixels, each consisting of three bytes, every byte can represent 256 intensity values for each primary color. A high degree of disorder in information is indicated by entropy values approaching 8, computed with Equation (3).

However, entropy alone is not a sufficient metric. It is conceivable to construct a histogram where all 256 intensities $x_t$ of a primary color occur with equal frequency, and therefore the same probability of occurrence $P(x_t)$ yet still exhibit a discernible pattern rather than randomness. In such cases, despite an entropy value of 8, the information cannot be classified as disorderly. This underscores the necessity of employing multiple parameters to assess the disorder level of pixels.

3 $$\begin{aligned} H(x)= -\displaystyle \sum _{t=0}^{255}P(x_t)\log _2P(x_t) \end{aligned}$$

#### Goodness-of-fit test

In this research, another method employed to measure the level of chaos in image information is the goodness-of-fit test using the chi-square $\chi ^2$distribution^28^. This tool operates as a hypothesis test, where the null hypothesis assumes that the distribution of the a histogram with the $n=$ 256 intensity levels adheres to a uniform distribution, implying equal *E* number of appearances for each intensity value $o_i$. Conversely, the alternative hypothesis posits the absence of uniformity^29^.

The test statistic $\hat{\chi }^2$ is defined by Equation (4) follows a $\chi ^2$ distribution with $n-1$degrees of freedom. However, due to the central limit theorem, this variable approximates a normal distribution^30^. For a histogram with $n=$ 256 bins, the mean $\mu = 255$ and the standard deviation $\sigma = 22.58$. Given a significance level of $\alpha = 0.01$, the decision rule is as follows: accept the null hypothesis if $\hat{\chi }^2 < 308$; otherwise, reject it.

4 $$\begin{aligned} \hat{\chi }^2= \displaystyle \sum _{i=1}^{255}\frac{(o_i-E)^2}{E} \end{aligned}$$

#### Discrete fourier transform

In the discrete Fourier transform, it is determined that the analyzed data contains no repeated bit strings. Furthermore, this method serves as a statistical hypothesis test where the null hypothesis declares that the image data lacks repetitions, implying it contains disorder or randomness^31^. Additionally, this test forms a component of the NIST 800-22standard^32^.

The test statistic $\hat{d}$ is defined by Equation (5), where *n* is the length of the bit string, $C_0$ is a constant defined in Equation (6), and $C_1$ is a variable. This variable $C_1$ represents the count of complex functions $f_j$ for which its modulus$\Vert f_j \Vert$ is less than the threshold *u*, as specified in Equation (7), with $j=1, 2, \ldots , n/2-1$. It is important to note that *n* is even, in this work, it represents the number of pixels multiplied by 24 to express it in bits.

5 $$\begin{aligned} \hat{d}= & \frac{C_1 -C_0}{\sqrt{\frac{n(0.95)(0.05)}{4}}} \end{aligned}$$

6 $$\begin{aligned} C_0= & \frac{(0.95) \times n}{0.05} \end{aligned}$$

7 $$\begin{aligned} l= & \sqrt{\text {Ln} \frac{1}{0.05}(n)} \end{aligned}$$

Regarding the function $f_j$, defined in Equation (8), it reads all bits of the string, with $y_k$ taking a value of −1 if the *k*-th bit of the string is 0, and 1 otherwise. Here, $i = \sqrt{-1}$ denotes the imaginary unit.

8 $$\begin{aligned} f_j = \sum _{k=1}^n y_k e^{\frac{2i\pi (k-1)j}{n}} \end{aligned}$$

Furthermore, if the *p*-value, as defined in Equation (9), is less than 0.01, the hypothesis of randomness is rejected at a significance level of 0.01; otherwise, it is accepted. The erfc function is defined in Equation (10).

9 $$\begin{aligned} & p-value = \text {erfc}\frac{\mid d \mid }{\sqrt{2}} \end{aligned}$$

10 $$\begin{aligned} & \text {erfc}\frac{\mid d \mid }{\sqrt{2}} = 2(1 - \Phi (\mid d \mid )) \end{aligned}$$

### Properties of the number pi

Under the experiment of the random selection of a bit $x_i$ from the digits on the right side of a decimal point of the number $\pi$, the probability of it being either zero or one is 0.5^33^. In other words, $p(x_i = 0) = p(x_i = 1) = 0.5$. In addition to the distribution of the digits from number $\pi$, the following two properties contribute to the seemingly chaotic appearance of these digits^34^.

1. The values of $\pi$ are deterministic. This means that for any given fixed block of positions, the values will always be the same.
2. For any sequence of digits in $\pi$, the values cannot be predicted unless one knows the exact position of the sequence and performs the extensive computations necessary to determine the sequence values^35^.

These characteristics support the proposal to use $\pi$ in digital signatures as part of cryptographic systems.

## Proposed digital signature

### A S-Box building from a permutation algorithm

In this section, we will outline the process of obtaining a permutation on an array of 256 elements, ranging from 0 to 255. It is noteworthy that a S-box of 8$\times$8, can be interpreted as a permutation of 256 elements^36^.

The current permutation algorithm is based on the factorial base representation of a number^37^, similar to other approaches^38^. The main difference is that our proposal has a time complexity of *O*(*m*), given that it only replaces values, this reduces the permutation process of *m* elements.

We initiate by defining the set $Z_m$ for $m \ge 2$ in Equation (11). The elements within this set are represented by indices denoted as *n*. Each index *n* signifies a specific permutation number within the *m*! permutations for an array of *m* elements.

11 $$\begin{aligned} Z_m = \{ n \in \mathrm I\!N | 0 \le n < m!\} \end{aligned}$$

Furthermore, for all number $n\in Z_m$, *n* can be expressed in a factorial basis, as shown in Equation (12).

12 $$\begin{aligned} n = D_0 (m-1)!+ D_1 (m-2)!+\ldots D_{m-2} (1)!+ D_{m-1} (0)! \end{aligned}$$

Where, according to the Euclidean division algorithm, the coefficients $D_i$ in Equation (12) are unique^39^. Furthermore, the constants $D_i$ satisfy the inequality of Equation (13).

13 $$\begin{aligned} 0 \le D_i < m-i \text { for } 0 \le i \le (m-1) \end{aligned}$$

With this previous concepts, the algorithm to generate a permutation of *m* elements is described below:

1. Propose randomly $m$ constants $D_i$ such that each satisfies Equation (13). Afterward, the permutation number $n$ to generate can be checked using Equation (12) by substituting the proposed $D_i$ constants.
2. An ascending array *Z* of *m* elements is constructed as follows: $Z[0] = 0, Z[1] = 1, \cdots, Z[m-1] = m-1.$
3. In the first iteration, the constant $D_0$ is selected. In accordance with Equation (13), $0 \le D_0 < m$, and consequently, $Z[D_0]$ is an existent element within the array generated in the previous step. Then, this study proposes that $Z[D_0]$ is the first element of the permutation *P*, it is $P[0]=Z[D_0]$, and to avoid repetitions, it is replaced with the last element of the array, $Z[m-1]$, for the following iteration. Additionally, the element $Z[m-1]$ is removed from the current array, resulting in an array of $m-1$ elements for the next iteration.
4. In the second iteration, the constant $D_1$ is selected. Following that, $0 \le D_1 < m-1$, consequently, $Z[D_1]$ is an existent element within the obtained array of $m-1$ elements in the previous step. Similarly, $Z[D_1]$ is now the second element of the permutation, and to avoid repetitions, it is replaced with the last element of the array, $Z[m-2]$, for the following iteration. Additionally, the element $Z[m-2]$ is removed from the current array, resulting in an array of $m-2$ elements for the next iteration.
5. In general, the constant $D_i$ is selected. Following that, $0 \le D_i < m-i$, consequently, $Z[D_i]$ is an existent element within the obtained array of $m-i$ elements in the previous step. Similarly, $Z[D_i]$ is now the *i*-th element of the permutation, and to avoid repetitions, it is replaced with the last element of the array, $Z[m-(i+1)]$, for the following iteration. Additionally, the element $Z[m-(i+1)]$ is removed from the current array, resulting in an array of $m-(i+1)$ elements for the next iteration.

As mentioned earlier, this algorithm has a time complexity of *O*(*m*) because only one elimination and one substitution are performed in each step. Also, if $Z[D_i]$ is the last element of the array in iteration *i*, then its position is taken by the immediately preceding element $Z[D_{i-1}]$. An exception occurs in the last iteration, where there will be only one element, and this will be the last element of the permutation.

To aid in understanding the proposed permutation algorithm, we provide an example of permuting an array with $m = 4$ elements, which helps clarify the process. This array allows for $4! = 24$ distinct permutations, spanning $n = 0$ to $n = 23$ within the set $Z_4$. By permuting these four elements, we can construct a S-box $2 \times 2$, which takes a 2-bit input and generates a 2-bit output. The S-box is arranged as a matrix with 2 rows and 2 columns, having $m=4$ elements; here, the first bit of the input (from left to right) designates the row, while the second bit designates the column to select the output element.

1. Propose randomly $m=4$ constants $D_i$ such that each satisfies Equation (13):
  - For $$i=0$$, $$D_0=2$$, satisfying $$0 \le D_0 < 4-0$$.
  - For $$i=1$$, $$D_1=1$$, satisfying $$0 \le D_1 < 4-1$$.
  - For $$i=2$$, $$D_2=0$$, satisfying $$0 \le D_2 < 4-2$$.
  - For $$i=3$$, $$D_3=0$$, satisfying $$0 \le D_3 < 4-3$$.
  Therefore, we will generate the permutation number $n = 14$, as can be seen in Equation (14), where *n* is expressed on a factorial basis with the proposed $D_i$ constants.
  14 $$\begin{aligned} 14 = 2(3)! + 1(2)! + 0(1)! + 0(0)! \end{aligned}$$
2. An ascending array *Z* of *m* elements is constructed: $Z= \{0, 1, 2, 3\}$
3. Iteration process
  - In the first iteration, $Z[D_0 = 2]$ is identified as the first element of the permutation $P$, specifically $P[0] = Z[2] = 2$. To prevent repetitions, $Z[2]$ is replaced with the last element of the array, $Z[3] = 3$. This results in the updated arrays:
    $$\begin{aligned} \displaystyle P = \{2\} \text { and } Z = \{0, 1, 3, 3\} \end{aligned}$$
    Finally, $Z[3]$ is removed, yielding the array $Z = \{0, 1, 3\}$.
  - In the second iteration, $Z[D_1 = 1]$ is identified as the next element of the permutation $P$, specifically $P[1] = Z[1] = 1$. To prevent repetitions, $Z[1]$ is replaced with the last element of the array, $Z[2] = 3$. The updated arrays are:
    $$\begin{aligned} \displaystyle P = \{2, 1\} \text { and } Z = \{0, 3, 3\} \end{aligned}$$
    Finally, $Z[2]$ is removed, resulting in $Z = \{0, 3\}$.
  - In the third iteration, $Z[D_2 = 0]$ is identified as the next element in $P$, specifically $P[2] = Z[0] = 0.$ To avoid repetitions, $Z[0]$ is replaced with the last element of the array, $Z[1] = 3$. The updated arrays are:
    $$\begin{aligned} \displaystyle P = \{2, 1, 0\} \text { and } Z = \{3, 3\} \end{aligned}$$
    Finally, $Z[1]$ is removed, yielding $Z = \{3\}$.
  - In the fourth iteration, $Z[D_3 = 0]$ is selected as the fourth element of $P$, specifically $P[3] = Z[0] = 3$. With $Z[0]$ now assigned, the final arrays are updated to:
    $$\begin{aligned} \displaystyle P = \{2, 1, 0, 3\} \text { and } Z = \{\} \end{aligned}$$
  Now, arranging $P$ as a S-box 2*x*2:
  $$\begin{aligned} \begin{bmatrix} 2 & 1 \\ 0 & 3 \\ \end{bmatrix} \end{aligned}$$

In this work, the constants $D_i$ are randomly chosen rather than specifying the number *n* directly. Expressing *n* in factorial basis to find the constants $D_i$ is avoided, as this process consumes more time, and one of the objectives of this research is to reduce computation time. Moreover, this tool will be utilized later to permute 256 elements and generate an S-box $8 \times 8$. As the constants $D_i$ are chosen randomly, the resulting permutation is also random, implying that the S-box $8 \times 8$will be dynamic. To conclude this section, it is emphasized that this algorithm generates a bijective function^40^.

### Parameters and keys generation

1. Prime *p*: The prime $p$ is defined as $p = n(q_1 \times q_2) + 1$, where $q_1$ and $q_2$are 1024-bit selected prime-numbers (verified previously using the Miller-Rabin algorithm^41,42^). While *n* is an even number, initially set to $n = 2$. The resultant value of $p$ is tested for primality using also the Miller-Rabin algorithm. If $p$ is not prime, $n$ is incremented to the next even number (e.g., $n = 4$), and the value of $p$ is recalculated. This iterative process continues, incrementing $n$ as needed until a prime $p$ is found that passes the Miller-Rabin test. Constructing $p$ in this manner ensures that $p - 1$ is divisible by $q_1$ and $q_2$, which allows us to know its prime factors directly, avoiding costly computational operations.
2. Generator $\alpha$: A candidate for the generator $\alpha$ is selected randomly such that $0< \alpha < p - 1$. $\alpha$ is validated as a generator if it satisfies the condition $\alpha ^{(p-1)/q} \pmod {p} \not \equiv 1$ for each prime factor $q$ of $p - 1$. If this condition is not met, $\alpha$ is incremented by one, and the verification is repeated for all prime factors $q$ of $p - 1.$ This process continues until $\alpha$ meets the condition. Note that the prime factors of $p - 1$ include $q_1$, $q_2$, and the prime factors of the chosen even number $n$ in the prime generation.
3. Hash function: The SHA3-512 function is selected^43^, denoted as *H*.
4. Private keys: Two numbers, denoted as $a_A$ and $a_B$, are selected as the private keys for users *A* and *B* respectively.
5. Public keys: The users possess public keys, denoted as $\beta _A$ and $\beta _B$, calculated as follows: $\beta _A = \alpha ^{a_A} \pmod {p}$ and $\beta _B = \alpha ^{a_B} \pmod {p}$.

### Procedure for signing a message

Below is the procedure for user *A* to sign the message *X*. Regarding the S-Box application, for each byte, use the first four bits (from left to right) to select the row and the remaining four bits to select the column of *S*, following the procedure similar to that of standard AES^44^.

1. Randomly select an integer *k*, such that $1< k < p-1$.
2. Compute the parameter $\gamma = k \times (\beta _B^{a_A}) \pmod {p}$.
3. Compute the Hash function *H* on the message *X* to obtain *H*(*X*).
4. Compute the product of $k \times H(X) \times \pi$ and extract the first 2048 bits to the right of the decimal point to create the string *C*.
5. The following 256 bytes of the previous product (after the 2048 bits previously used) are selected to form an array *d* with 256 elements $d_i$, each of one byte in size. This implies that $0 \le d_i \le 255$.
6. Compute 256 constants $D_i$ as $D_i = d_i \pmod {256-i}$ for $0 \le i \le 255$ and apply the algorithm described in Permutation algorithm to obtain the substitution box *S*.
7. Compute the parameter $\delta =S(C)$, by applying the S-Box *S* to the string *C* in one-byte blocks.
8. From this point, A’s signature would be $Sig( X, k) = (\gamma , \delta )$.

### Procedure for verifying a sign

Below is the procedure for user *B* to verify the signature of user *A*. The session key $\beta$ of the Diffie-Hellman protocol is defined as $\beta = \beta _B^{a_A} \pmod {p} = \beta _A^{a_B} \pmod {p}$, and is known only by both users. Its inverse, $\beta ^{-1}\pmod {p}$ always exists. If the public keys $\beta _A,\beta _B$ remain unchanged for every message, the $\beta ^{-1}\pmod {p}$ computation can be performed by both users prior to the communication. If the message *X* is altered, the output of the Hash function may differ from the original, which is denoted as $H'(X)$ instead of *H*(*X*). This notation is similarly applied to subsequent calculations that may also be affected.

1. Once *B* has received the parameter $\gamma$, the value of *k* is computed as $k = \gamma \times [(\beta _A)^{a_B} ]^{-1} \pmod {p}$.
2. Compute the Hash function *H* on the message *X* to obtain $H'(X)$.
3. Compute the product of $k \times H'(X) \times \pi$ and extract the first 2048 bits to the right of the decimal point to create the string $C'$.
4. The following 256 bytes of the previous product are chosen to create an array $d'$ with 256 elements, each of one byte in size. This implies that $0 \le d'_i \le 255$.
5. Compute the constants $D'_i$ as $D'_i = d'_i \pmod {256-i}$ and apply the permutation algorithm described in the previous subsection to obtain a permutation of 256 elements and, consequently, the substitution box $S'$.
6. Compute the parameter $\delta '=S'(C')$, by applying the S-Box $S'$ to the string $C'$ in one-byte blocks.
7. The verifier B can verify that A signed the message if $\delta '=\delta$.

**Fig. 1:**
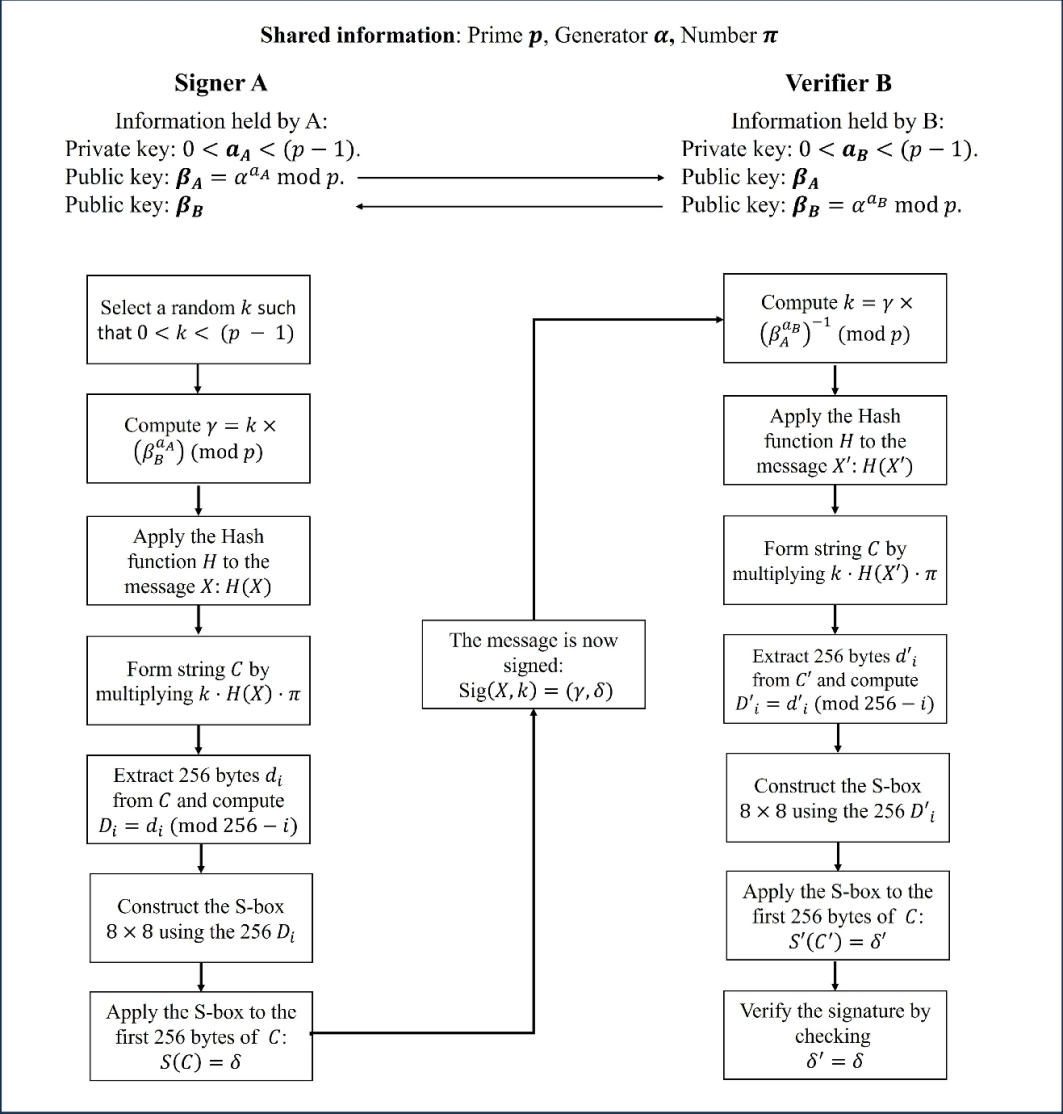
Diagram of the proposed DSADH$\pi$ scheme, illustrating the procedures for message signing by the signer and signature verification by the verifier.

Additionally, to clarify the steps outlined in the Procedure of signing a message (conducted by the signer, A) and the corresponding steps of the Procedure for verifying a sign (performed by the verifier, B), both processes are illustrated in Figure 1. This figure provides an overview of all elements and steps involved in the proposed method.

## Experimentation and results

In this research, a procedure is proposed to assess the quality of the presented signature when only the private key $a_A$ is varied while keeping the message constant. The proposed method involves creating a color image of 512 $\times$ 512 pixels with each resulting signature. This image is constructed by concatenating the strings that conform to the parameters $\gamma$, and $\delta$. Subsequently, entropy and correlation metrics are applied to the generated images.

Additionally, these steps facilitate a comparative analysis of the DSA and the proposed method. This comparison is achieved by varying only the message *X* in both algorithms while keeping all other parameters constant. For this study, a signature algorithm is considered superior if the entropy of the generated image is closer to 8, and the correlation is closer to zero. The parameters used in the experimentation are presented in Table 1, including the prime number *p* and generator $\alpha$. Table 2 lists the public and private keys of the signer and verifier, along with the associated session key.

**Table 1:** Parameters utilized in reproducing the Proposed Algorithm for the experimental results. The prime number $p$ and generator element $\alpha$ were computed as outlined in the Parameters Generation subsection.

Variable	Hexadecimal value
*p*	47a9c62c 3021e4db d0e8ee54 8d5e1b2d c6a1c4f5 bb0345f7 20ba54f2 256a9bef
	b394d7b1 55fa27bf e1701aa3 f61732f8 52c51dee ebd0b20b fc098266 348d5cdf
	f871e3d4 a6b623a3 a40c3a44 95d13b69 9771c4c9 d036ef1c ce70c746 d618ad20
	00520ffe 9db5764e 74e2344b efe45688 d4a4ff14 219e346d e9911f39 8aa6c864
	38e38ab0 ffcfe561 60fb060f 361e0c06 335c458a 516a6bd6 a0e42677 593be5b0
	d6639051 52c4ffb9 d0cbc755 2904a7c1 34530089 50343bd2 04d41ed3 d47cd4bf
	4fa03956 208dfe0c 5f1d9fa8 27507507 3ca63f20 df1de1bb edec281f 890956a2
	355e8517 a334e9c1 e8c8aea8 62d3b596 5e1c6c22 ef7efe21 2154c979 e9fbdfc7
	e7445f
$\alpha$	1bc36c7f d8704a7d 008f3200 70dc9c2a 2cd51047 d7d48401 816693f6 df3c83f6
	a99d72bf 3d8555f1 92dcbe33 aaabad16 4070b905 b92a8d2b bc8f2c72 eab5bb61
	fa1e5493 c21c7fd1 dd8c2e50 f06072b4 c31bf0e3 ca3e0757 4c361d6b 9eec4742
	29ae024f b3fcf3f0 40f937ee f2e6383e 644189be 811aaf8e 7175111c e364a24c
	960e7273 514b7772 fffaa39d 7074465e 53a76ec7 8ac3270a 938295fc f51b5dc9
	62240658 513aaab0 c383005d bf7ae530 c5fcd4c7 420e0301 bb4d89b0 e3de8f61
	f6c84415 5c81aed6 18c6dd7e 4be4d227 7376e6b9 3c4001a7 17b23769 11f14a01
	7caf3d6f 8da8adab eeed578d 0fa3a4bd 1b7b74ad be82b232 7936f352 b60248ce

**Table 2:** Public, Private, and Session Keys.

Variable	Hexadecimal value
$a_A$	c3ef3be7 248d72be 5de8d531 ff2b5aaa 1c5c6caf 2d7fade9 4302fd3e b923f53e
	0e9fa67a 15fa19a8 42af51dd b4a6d897 fe633c9c 5c908cfeca 68df86fe 58cdcc08
	300cc05e a38c0020 cb8d6d19 a465a768 8d97122c ca67a978 fcd71d1c c709c96a
	0213d389 753cfa8a 99d212a6 48e1a3aa 18bf8603 6ebbe10e eed469a9 50163c74
	572d00e1 fdb9fbbc 08531b34 86ec9d0c 46f25c95 43cf5eb4 d31d2071 adaf339a
	6ee586e9 38c0e376 2a449202 9ffbd536 a98d77a3 f63b740f 7aea002b 0f684481
	8863da1f 7f14ae89 6ed73550 2fb18b2f 77818ceb a6e6d4be 952dc9bb 37e6b6fc
	e38ea1f7 f174ee80 04590ea1 a22600a0 e2a8a584 d9f40273 c2e73d82 e5284a
	4067213f 39fd257d 30ac2a17 470628c0 beb1dc53 c806ce46 32e026e4 350299cc
$\beta _A$	7f484072 af98a7f8 8e67cbee 75ea7721 c09a7285 08dd7626 438ad5d4 9c7716f6
	d5d397ce c0c7b98f 22948cec 6b251e19 8ea11c48 690320d8 faa22cc2 0ee21fc7
	6eb50ed8 4f226d13 536fecb7 d85520d4 e78eb36d ed395ca0 7d3ef85c 906e8a59
	2042e948 fcfdc3c7 d78d79e8 bccb577c 9c00796c 47421215 bd9c0fb8 b03a41e4
	901e6526 42b2d63e 0885c2c1 61e9da36 c10d363f 848ec2d9 73a5dc15 5e9129c7
	3adb1411 920a72f3 653c2384 5558805a 9d404e71 16a8be0b bbad58c2 837520a5
	b4220e16 d27aa628 a1ec3f19 1363db1d b54380d8 7480b2ee 48bc1580 c06a26a2
$a_B$	c4b942d9 c75e9504 95fa22f4 6b196ecc c45fb533 2fa4b4b2 3ffff999 144af53c
	80f08ea8 9c639167 7d8da425 8938cb48 dbf085ca 2bbbb989 9725c4ee 9dc4e47c
	e7c1e80b 0e1f2825 cd97540e b8bac8a9 07fe02eb 404a93c4 4b065bc4 7489fb10
	ede073e0 e838d910 f4375ea9 5fd462f1 4bd8e787 2a3acf19 da600f26 c9a62ac0
	aa6371c6 33234648 814db059 be90be45 9c991d52 6aa04476 651352ee 84059f89
	83acfcc9 2936dcac fe2264d1 c0763e7a 05397292 c22e1025 df53a4ef 69542ab6
	8bc4ae10 73cf47a6 e42cad7a eff27ad8 1fa94c5a fabcd543 068f43fd 25de8248
	237a3357 84d82935 4d541ac2 89cb23cf 9166b78a 6c648d29 bd09703d 50edbf4a
$\beta _B$	25df467d 93e9ad76 0aadaeab f23be2bb b6fd5797 07964944 33ed719b ac3878ca
	b157ee2b 05f52094 75ce133c eab202f8 cbae552f 62852bda 4031f160 3cc08213
	4a111fcf 3a8867e2 c5ab884b 9da0b6a9 fe1bc9ec e7410467 421e03cd e66d5a2d
	e2840c88 96c1eb63 d0230c17 ac46af28 4e1d15de 080004fa 87b7cd81 6c5c09d4
	4295e385 eca13f00 659a1532 de359a7d 7716032d b3998b5a db0b529f 36a6d5c6
	5f8c831e 555f17de 40f84120 35745986 52568a39 9f159327 fc1da17a 75b2dfa0
	87c9ec69 bcd09efe 897f4acf 41c8c6fc 93ecbf d7ad60c7 344e8451 c8dbdad5
	b51544ed 769
$\beta$	154b4820 e5d1480d 15f78ae7 1061a269 99c9bce5 883f22b7 0083ccad cc15b035
	92ab8946 17408604 87fb8c8b 49c785f1 b68a2884 b0072839 21a0531d c79f5e21
	2bc27fd0 500855c3 2942fd75 9bb491f0 24133ee6 928fca81 16a68690 0004177f
	a04b4ae2 ddc7b504 49abcf17 c421b8ea 30f67173 6771d257 94bdfde4 2f2cedd0
	d2378942 150023a2 d1cc9497 93ec7ee2 172277be dc4a27cf 50c9352b c3b1a512
	0dfd55a3 778604c ac6f1b52 350909d6 2d9e2e26 3c81b5b3 a96f7cf5 2efbaf73
	8776138b 454fd32d 3fe6fa50 1ad84ae2 f70a2b6c 97caee23 446feede 85dd58a5
	f3a62b7c 965cc78e 6e551d34 d50eddfc 9f756e97 04345edf 4bf15c6e 9b03f320
	b1f2

### Private key sensitivity

**Fig. 2:**
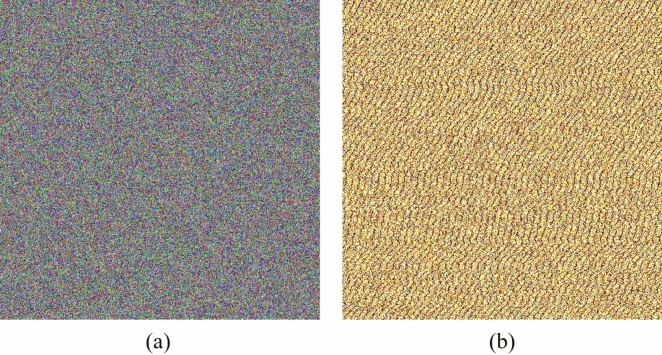
Images generated by concatenating the parameters $\gamma$ and $\delta$ of DSADH$\pi$ signature after private-key modifications. (a) Image generated using DSADH$\pi$ signatures. (b) Image generated using DSA signatures.

The authentication property of the proposal is verified by testing two aspects: first, showing that any alteration in the signer’s private key $a_A$ produces a distinct signature $(\gamma , \delta )$ for the same message *X*, value *k*, and public key $\beta _B$; and second, ensuring the lack of correlation between them. To perform this, the following experiment is led: different signer’s private keys are proposed, and the resulting signatures from each key modification are concatenated into a byte string to conform an image. These outcomes are depicted in Figure 2, Figure 2(a) is composed with signatures from DSADH$\pi$, and Figure 2(b) from DSA. While the assessment of information disorder is conducted by evaluating entropy, correlation, discrete Fourier transform, and goodness-of-fit parameters as outlined in the Table 3, where the $\checkmark$ indicates that the test has been passed. Otherwise, $\times$ means that the test has not been passed.

**Table 3:** Encryption measures for the images constructed from DSADH$\pi$ and DSA signatures using different private keys.

Measure	Figure 2(a)			Figure 2(b)		
	Red	Green	Blue	Red	Green	Blue
Entropy	7.9992	7.9993	7.9992	3.3388	5.2254	7.8299
Horizontal correlation	−0.0161	0.0117	−0.0009	0.0355	0.0277	−0.0007
Vertical correlation	−0.0143	−0.0135	−0.0041	0.0715	0.0676	0.0512
Diagonal correlation	0.0049	−0.0119	0.0036	0.0249	0.0356	0.0275
$\hat{\chi }^2$	276.8$\checkmark$	250.5$\checkmark$	285.5$\checkmark$	30033349.9$\times$	13144946.2$\times$	136694.2$\times$
DFT *p*-value	0.2380$\checkmark$	0.6811$\checkmark$	0.8878$\checkmark$	0.0$\times$	0.0$\times$	0.0$\times$

### Message Sensitivity

**Fig. 3:**
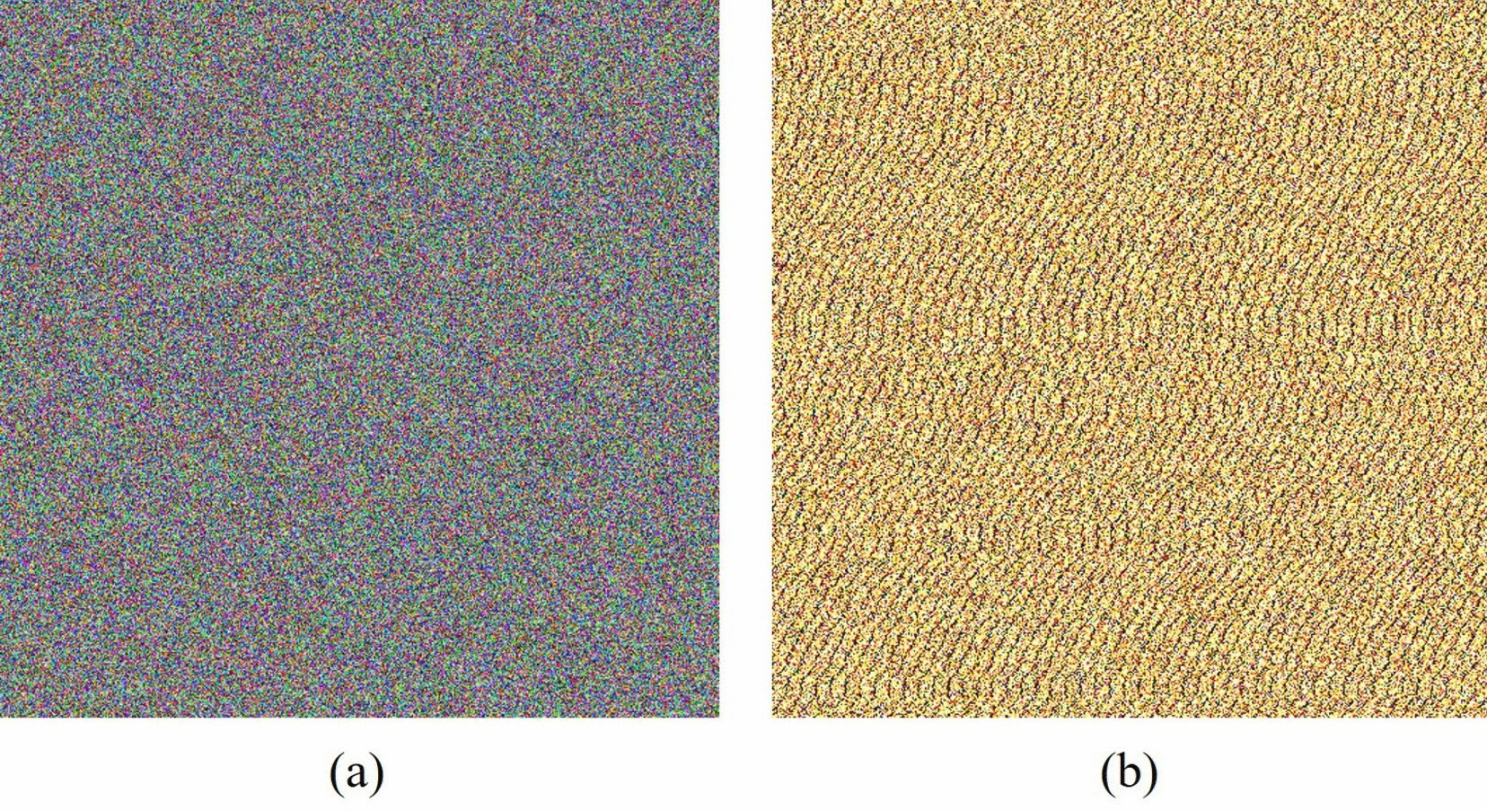
Images generated by concatenating the parameters $\gamma$ and $\delta$ after message modifications. (a) Image generated using DSADH$\pi$ signatures. (b) Image generated using DSA signatures.

Regarding the integrity property, it is verified that the proposed signing algorithm detects changes in the message *X* through the following experiment: randomly selecting different messages *X* that are signed using the same private $a_A$ and public key $\beta _B$, along with the same value *k*. Specifically, step 3 of the signing algorithm involves applying the SHA function *H* to each different message *X*. Subsequently, the multiplication result of step 4 is distinct for each message, altering the outcome of subsequent steps, resulting in different values of $\gamma$ and $\delta$. These sequences are concatenated to generate Figure 3(a). Additionally, the same procedure is applied to DSA, with Figure 3(b) displaying the image produced by concatenating the resulting signatures. The results are presented in Table 4.

**Table 4:** Encryption measures for the images constructed from DSADH$\pi$ and DSA signatures using different messages.

Measure	Figure 3(a)			Figure 3(b)		
	Red	Green	Blue	Red	Green	Blue
Entropy	7.9993	7.9992	7.9993	3.3337	5.2380	7.8304
Horizontal correlation	−0.0009	−0.0118	−0.0039	0.0320	0.0128	−0.02334
Vertical correlation	0.0022	−0.0151	−0.0063	0.0721	0.0606	0.0386
Diagonal correlation	−0.0071	−0.0053	−0.0032	0.0183	0.0166	0.0140
$\hat{\chi }^2$	265.2$\checkmark$	286.6$\checkmark$	243.6$\checkmark$	30088421.4$\times$	13050060.0$\times$	136386.5$\times$
DFT *p*-value	0.7839$\checkmark$	0.9535$\checkmark$	0.4768$\checkmark$	0.0$\times$	0.0$\times$	0.0$\times$

## Results analysis and discussion

We explore the security aspects to both the DSA and the DSADH$\pi$ proposal. A common vulnerability is the risk of an attacker deducing the signer’s private key from the public key, which would enable them to sign messages and forge the signer’s signature. This threat stems from the Discrete Logarithm Problem, which is referred due to the way public keys are generated. Research into this issue focuses on attacks with a computational complexity of $O(\sqrt{p})$^45^.

Given that the DSADH$\pi$ operates with a modulus $p \approx 2^{2048}$, the complexity of such attacks is $O(\sqrt{p}) \approx 2^{1024}$. Due to the proposed size of the prime *p*in this study, executing such attacks successfully is currently infeasible. Additionally, when employing the Diffie-Hellman protocol, there is the risk of a man-in-the-middle attack. However, this risk can be mitigated through effective key management strategies facilitated by cloud computing^46^.

Regarding the computational complexity involved in executing the DSA and DSADH$\pi$ algorithms, a notable distinction is that the DSADH$\pi$ proposal does not require the calculation of an inverse modular multiplicative each time a message is signed. This feature eliminates the risk associated with the integer *k* in the DSA algorithm, where there is a possibility that *k*, generated at random, may not have an inverse, necessitating its regeneration and additional execution time. In contrast, the DSADH$\pi$ proposal allows any value of *k* to be used, streamlining the signing process.

On the other hand, a closely related approach to our proposal, as it also incorporates a substitution box for the signature process, is the algorithm proposed by Hematpour et al^13^. The primary distinction between their method and ours lies in their use of a static S-box. Dynamic S-boxes offer superior entropy, making the system more resilient against linear and differential attacks^47^. The trade-off for using dynamic S-boxes is the additional computation time required to generate a new one for each signature; however, this increase in security justifies the added computational cost. Additionally, despite the time needed for computing dynamic S-boxes, DSADH$\pi$ still performs faster than DSA in signature generation.

Furthermore, the proposed method considers large-scale applications, given the number of unique signatures that a signer can generate. Specifically, the total number of possible signatures arises from the values produced by the product $k \times H(X)$ in step 4 of the Procedure of signing a message, which is used for constructing the string *C* and, consequently, the signature itself. Here, the value *k* has $2^{2048}$ different values, while the SHA function used can return $2^{512}$ different strings, resulting in a total of $2^{2048} \times 2^{512} = 2^{2560}$ distinct combinations. This quantity indicates the number of unique signatures that can be generated by a signer.

Regarding the security, in study, two color images of 512 $\times$ 512 pixels were generated by concatenating the bit strings $\gamma$ and $\delta$ obtained from signing different messages *X*. One image was created using the DSA algorithm, while the other was generated using the proposed DSADH$\pi$. The algorithms were implemented in Java and executed on a computer equipped with an i9-10900K processor running Windows 11. The time required to build the image with the DSA algorithm was 3964.6649 ms seconds, compared to 87.8547 ms seconds for the proposed algorithm. Thus, the proposed algorithm achieves superior speed compared to DSA due to the elimination of inverse calculations.

In addition, an evaluation with encryption measure of the signatures was conducted, as shown in Tables 3 and 4. DSADH$\pi$ achieved entropy values above 7.999, nearing the optimal value of 8.0, while DSA showed entropy values of 3 in the red channel, 5 in green, and a maximum of 7.8 in the blue channel. This difference is visible in Figures 2(b) and 3(b), where DSA signatures exhibit a visual pattern with dominant colors. Although DSA achieved favorable correlation values close to the ideal of 0.0 (e.g., 0.0128), DSADH$\pi$ obtained similarly close values, including some even nearer to 0, such as −0.009, indicating the absence of a linear relationship between adjacent bytes.

However, correlation alone does not fully confirm randomness. A more significant difference between the two signatures is visible in the Goodness-of-fit test, where DSA produced values up to 8 decimal digits on the statistical test $\hat{\chi }^2$, far below the maximum optimal-value of 300 and achieved by DSADH$\pi$. The DFT test further underscored these differences: while the hypothesis of randomness is rejected if the *p*-value is less than 0.01, DSA consistently showed p-values of 0.0, rejecting randomness in all colors. In contrast, DSADH$\pi$ maintained values above the threshold, supporting the randomness hypothesis. Based on these encryption metrics, DSADH$\pi$ shows stronger security-properties than DSA.

## Conclusion

In this research, a digital signature algorithm is introduced named DSADH$\pi$, which integrates a dynamic substitution box to enhance both speed and security compared to DSA. The DSADH$\pi$ algorithm improves time efficiency by eliminating the step of computing a modular multiplicative inverse for every message signed. This approach permits the use of any integer *k*, avoiding the need to regenerate *k* if it lacks an inverse. For instance, when constructing a 512 $\times$ 512 pixels color image by concatenating the bit strings $\gamma$ and $\delta$ from signatures of different messages, the DSADH$\pi$ algorithm completed the task in just 87.8547 ms, whereas the DSA algorithm required 3964.6649 ms, the results are illustrated in Figures 2 and 3. In terms of security, the proposed algorithm demonstrates superior performance. As detailed in Tables 3 and 4, the proposed algorithm achieves higher security metrics: its entropy approaches 8, and its correlation is closer to 0, indicating greater robustness than DSA. In addition, the proposed method enables a signer to generate up to $2^{2560}$ distinct signatures. The DSADH$\pi$ algorithm demonstrates resilience against discrete logarithm attacks as well as cryptographic attacks typically associated with static S-boxes, due to its use of dynamic substitution boxes. These features collectively confirm the enhanced security of the approach.

## Data Availability

The digits of number $\pi$ used in this work are from: https://storage.googleapis.com/pi100t/index.html.
